# Denosumab combined with radiotherapy as an alternative to surgery for advanced metastatic bone lesions and pathologic fractures: a retrospective case study of 38 patients

**DOI:** 10.2340/1651-226X.2024.40977

**Published:** 2024-12-01

**Authors:** Mehdy Farhang, Martin Isaksson, Johan Wänman, Richard Löfvenberg, Sead Crnalic

**Affiliations:** Department of Diagnostics and Intervention, Orthopedics, Umeå University, Umeå, Sweden

**Keywords:** Denosumab, radiotherapy, pathologic fracture, impending fracture, metastatic bone lesion, bone metastases, non-surgical treatment

## Abstract

**Background and purpose:**

Pathologic and impending fractures occur in patients with advanced metastatic disease and necessitate surgical interventions with high risk of complications. The aim of this study was to analyze the efficacy of combined treatment with denosumab and radiotherapy as an alternative to surgery in treating bone metastases of the pelvis and extremities.

**Methods:**

This retrospective cohort study included 38 patients with impending and pathologic fractures due to carcinoma metastases who received monthly injections of denosumab (120 mg/dose) and radiotherapy. Twenty-three patients received denosumab and single-dose radiotherapy of 8 Gy, and 15 patients received denosumab and fractionated radiotherapy. We assessed pain, radiographic signs of fracture healing, survival and complications.

**Results:**

Of the 38 patients 36 experienced pain reduction. Callus formation was observed in 11/17 patients with pathologic fractures, and increased mineralization was found in 12/21 patients with impending fractures. In 23/38 patients, we found both pain reduction and callus formation or increased mineralization. There were no statistically significant differences in treatment outcomes between the patients who received denosumab and single-dose radiotherapy and those who received denosumab and fractionated radiotherapy. The survival rates at 30 days and 1 year were 95% and 56%, respectively.

**Interpretation:**

Combined treatment with denosumab and radiotherapy may reduce pain and promote bone healing in patients with metastatic impending and pathologic fractures. In this combined treatment, the effect of single-dose radiotherapy appears to be comparable to that of fractionated regimens.

## Introduction

Bone metastases can cause pathologic and impeding fractures, pain, spinal cord compression and hypercalcemia [1, 2]. Treatment of bone metastases is primarily palliative and aims to reduce pain, enable mobilization and improve quality of life [3]. Pathologic and impending fractures are usually treated surgically [4]. Surgery in these patients is associated with long hospital stays, increased risk of complications and mortality [5, 6]. When a tumor metastasizes to bone, complex interactions between cancer cells and osteoblasts and osteoclasts lead to an osteolytic, osteoblastic or mixed bone response [7], which increases the susceptibility of the bone to fracture. Activated osteoclasts play a central role in the bone destruction process in metastatic bone lesions [8]. Denosumab is a monoclonal antibody against receptor activator of nuclear factor-kappa B ligand (RANKL) that inhibits osteoclast maturation and bone resorption, which can result in bone formation [9]. Denosumab has shown efficacy in reducing the incidence of skeletal- related events in patients with bone metastases [10, 11]. In a systematic review in 2016, Gruenen et al. reported that there is a lack of evidence that radiotherapy, with or without bisphosphonates or RANKL inhibitors, increases bone density and prevents pathologic fractures [12]. Recently, a greater response rate was reported when bisphosphonates or RANKL inhibitors were combined with radiotherapy compared to single therapy with bisphosphonates or RANKL inhibitors in patients with osteolytic bone metastases from breast cancer [13]. The different mechanisms of action of RANKL inhibitors and radiotherapy may have synergistic effects on bone metastasis, but it is not known whether they provide sufficient bone strength to serve as a non-invasive alternative to surgery for pathologic and impending fractures. We aimed to evaluate the efficacy of combining denosumab with radiotherapy for reducing pain and improving bone healing in patients with impending or pathological fractures due to carcinoma bone metastases.

## Patients and methods

### Study design and setting

This is a retrospective cohort study that included patients over 18 years of age with carcinoma bone metastases causing pathologic and impending fractures in the extremities and pelvis who were treated between January 2014 and December 2022 at the Department of Orthopedics, Umeå University Hospital, Sweden. Patients with pathological hip and femur fractures who underwent emergency surgery were excluded. All patients received monthly injections of 120 mg of denosumab (XGEVA, Amgen, CA, USA) subcutaneously in combination with radiotherapy. Twenty-three patients received denosumab and single-dose radiotherapy of 8 Gy, and 15 patients received denosumab and fractionated radiotherapy (median dose 24 [interquartile range [IQR]: 20–25] Gy, median number of fractions 5 [IQR: 5]). None of the patients were receiving bisphosphonates at the time of referral. The first dose of denosumab was given at a median of 0 days (IQR: 11 days before – 18 days after) relative to the first dose of radiotherapy for the fractionated group and at a median of 2 days (IQR: 21 days before – 20 days after) for the single-dose group.

### Data collection and follow-up

Patient data included age, sex, type of primary tumor, skeletal location of metastatic infiltration or pathological fractures, pain before and after treatment, doses and fractions of radiotherapy and denosumab, adverse events, need for converting the therapy to surgical stabilization, and the date of death. Survival was recorded from the date of the first denosumab injection until 31 December 2023.

### Radiographic assessment

Pathologic fracture was defined as a visible fracture line in the tumorous area. An impending fracture was defined according to Harrington’s criteria as a lytic lesion larger than 2.5 cm, more than 50% cortical destruction and consistent pain in the tumorous area despite previous radiotherapy [14]. Two of the authors (MF and MI) assessed the radiographic response. Healing was defined as the development of a bridging callus for pathologic fractures and as increased mineralization in impending fractures.

### Evaluation of pain

The evaluation of pain was categorized as no pain, mild pain, moderate pain or severe pain before and after combined treatment (Table 1). A reduction in pain was classified as a transition from severe to moderate, mild or no pain after treatment.

**Table 1:** Definitions of pain categories.

No pain	No pain during activity or rest No use of pain medication
Mild pain	No pain during rest, pain during activity Use of non-opioid pain medication
Moderate pain	Pain during rest and activity Use of non-opioid and opioid pain medication
Severe pain	Pain during rest and activity Use of non-opioid and opioid pain medication with moderate effect

### Ethics

The study was conducted in accordance with the ethical principles of the Helsinki Declaration and was approved by the Swedish Ethical Review Authority (Diary number: 2023- 03833-01).

### Statistics

Descriptive statistics for continuous variables are presented as medians with interquartile ranges (25^th^ percentile to 75^th^ percentile = IQR) for non-normally distributed variables. Categorical data are presented as numbers. Differences in outcomes between types of radiotherapy, types of metastatic lesions, primary tumors, and skeletal segment locations were compared with a chi-square test. A paired comparison for different categories of pain before and after treatment was performed with the Wilcoxon signed rank test. Survival was estimated with the Kaplan-Meier method. A *P*-value ≤ 0.05 was considered to indicate statistical significance. The statistical package SPSS (IBM SPSS Statistics for Mac, Version 26.0, Armonk, NY: IBM Corp. USA) was used for the statistical analyses.

## Results

The study included 38 patients – 18 women and 20 men – with a median age of 71 (IQR: 62–78) years. Eleven different types of primary carcinomas were included. The most frequent origins were the kidney (*n* = 9), breast (*n* = 8), prostate (*n* = 7) and lung (*n* = 6). We stratified the types of lesions into pathological fractures (*n* = 17) and impending fractures (*n* = 21). The anatomical locations were the upper extremity (*n* = 14), lower extremity (*n* = 12) and pelvis (*n* = 12) (Table 2). Radiologically, 32/38 patients had lytic lesions and 6/38 had mixed osteolytic/osteoblastic lesions.

**Table 2:** Clinical characteristics of patients with bone metastases who were treated with denosumab (120 mg) and single-dose (8 Gy) radiotherapy or fractionated radiotherapy.

	Denosumab + single dose RT	Denosumab + fractionated RT
Number of patients	23	15
Female/male	13/10	5/10
Median age	71 (IQR: 62–70)	66 (IQR: 61–72)
**Primary tumor** Breast	6	2
Kidney	3	6
Prostate	5	2
Lung	4	2
Bladder	1	1
Colorectal	0	1
Liver	1	0
Thyroid	1	0
Melanoma	1	0
Endometrial	1	0
Esophagus	0	1
**Type of metastatic lesion** **Pathological fracture**	**12**	**5**
Upper extremity	8	3
Lower extremity	1	0
Pelvis	3	2
**Impending fracture**	**11**	**10**
Upper extremity	1	2
Lower extremity	9	2
Pelvis	1	6

RT: radiotherapy; IQR: interquartile range.

### Pain reduction

Of the 38 patients 36 experienced pain reduction (Table 3). A reduction from severe to mild or no pain was noticed in 30/38 patients. A reduction from severe to moderate was observed in six patients and no reduction in pain was detected in two patients. Pain reduction was observed in 22/23 patients who received denosumab in combination with single-dose radiotherapy and in 14/15 patients who received denosumab combined with fractionated radiotherapy.

**Table 3:** Radiological and clinical outcomes in patients with bone metastases after treatment with denosumab (120 mg) and single-dose radiotherapy (8 Gy) or fractionated radiotherapy.

Type of metastatic lesion		Outcome		
Pathological fracture		Callus formation	Pain reduction	Both callus formation and pain reduction
Denosumab + single dose RT	12	8	11	8
Denosumab + fractionated RT	5	3	4	3
Impending fracture		Increased mineralization	Pain reduction	Both increased mineralization and pain reduction
Denosumab + single dose RT	11	6	11	6
Denosumab + fractionated RT	10	6	10	6

RT: radiotherapy.

Of the 17 patients with pathologic fracture 15 experienced pain reduction compared to 21/21 patients with impending fractures (*P* = 0.19). There were no statistically significant differences in pain reduction regarding the anatomical location of the lesion (upper extremity 11/14 versus pelvis 10/12 versus lower extremity 9/12, *P* = 0.89).

14/38 patients experienced pain reduction without radiological healing; eight of them received denosumab and single-dose radiotherapy, and six received denosumab combined with fractionated radiotherapy. We found no signs of radiological healing in those two patients who experienced no pain reduction after treatment.

### Radiographic assessment: Callus formation/increased mineralization

**Figure 1:**
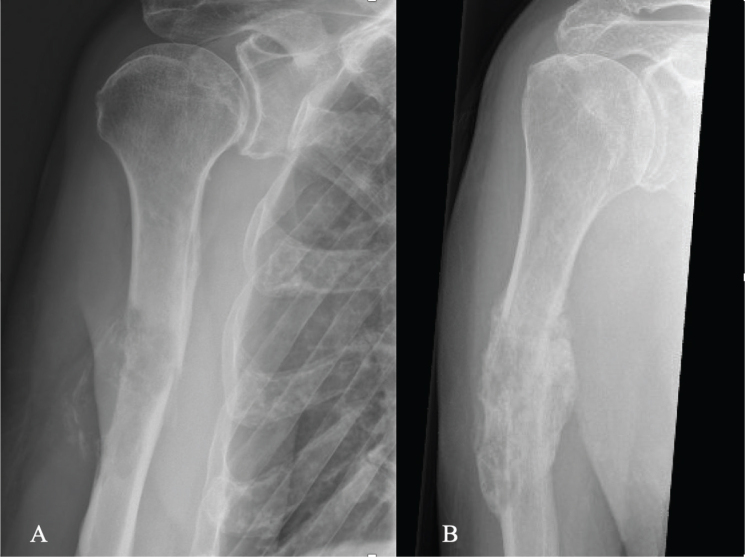
An 80-year-old man with kidney cancer and a pathological fracture in the right humerus (A). He was treated with denosumab and fractionated radiotherapy. Two months after the start of treatment, he had no pain. Bridging callus was observed 5 months after the start of treatment (B).

**Figure 2:**
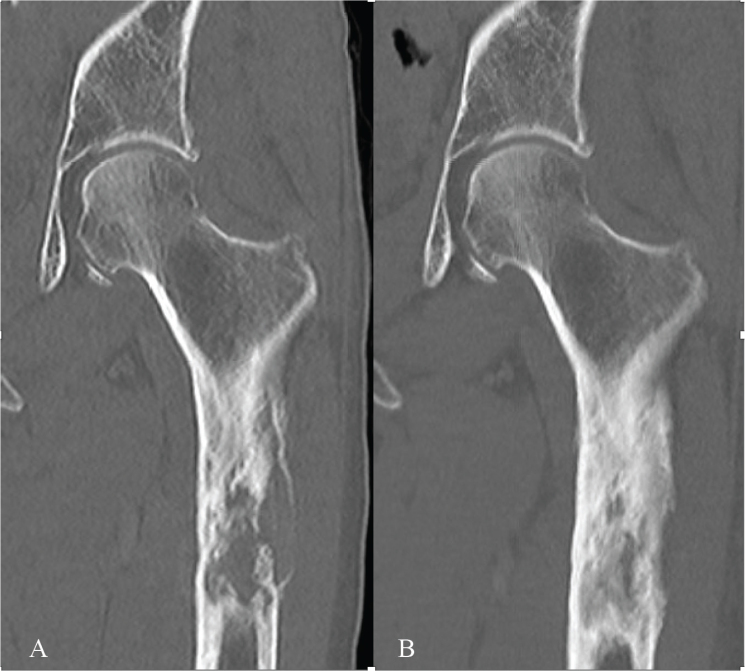
A 76-year-old woman with lung cancer and severe pain due to an impending femoral fracture (A). She was treated with denosumab and a single dose of 8 Gy radiotherapy. After 2 months, her pain was relieved. Increased mineralization was observed 6 months after the start of treatment (B).

**Figure 3:**
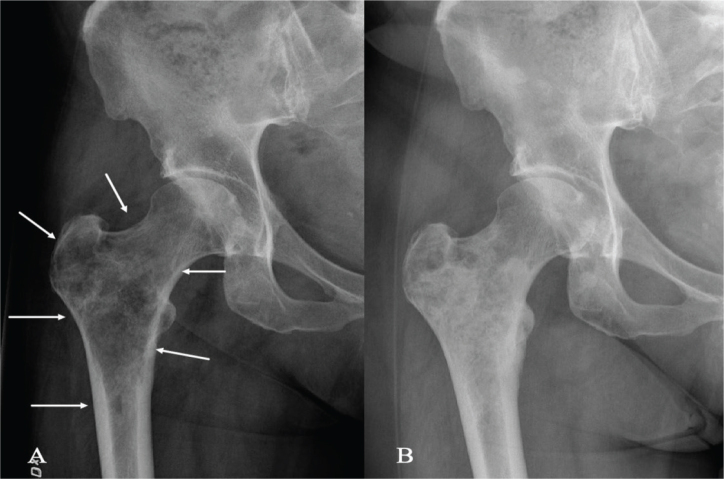
A 62-year-old woman with breast cancer bone metastasis in the right trochanter region causing severe pain (A). She was treated with denosumab and a single dose of radiotherapy of 8 Gy. After 2 months, she had no residual pain and increased mineralization was observed. One year after the start of therapy, the mineralization covered the entire metastatic inter- and sub-trochanteric areas (B). She had no pain and could walk without restrictions.

**Figure 4:**
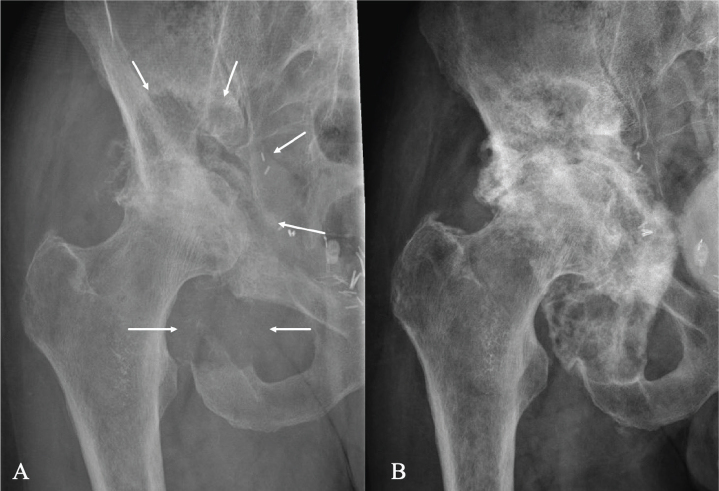
An 82-year-old man with prostate cancer and pathological fractures in the pelvis with protrusion of the femoral head (A). He had severe pain and was unable to bear weight on the right leg. He was treated with denosumab and a single dose of radiotherapy of 8 Gy. Radiography 8 months after the start of treatment revealed increased mineralization (B) and the patient was able to put full weight on his right leg.

Callus formation was observed in 11/17 patients with pathologic fractures, whereas increased mineralization was found in 12/21 patients with impending fractures (Figures 1–4, Table 3), with no statistically significant differences regarding the type of lesion (pathologic fracture 11/17 versus impending fracture 12/21, *P* = 0.45) or the anatomical location of the lesion (upper extremity 10/14 vs. pelvis 7/12 vs. lower extremity 6/12, *P* = 0.53). Callus formation was observed in 14/23 patients who received denosumab combined with single-dose radiotherapy and in 9/15 patients who were treated with denosumab combined with fractionated radiotherapy (*P* = 0.61).

Radiographic healing rates for the most common cancer types in our series were 4/9 for kidney cancer, 8/8 for breast cancer, 6/7 for prostate cancer, and 2/6 for lung cancer.

### Pain reduction with concomitant callus formation/increased mineralization

In 23/38 patients, both pain reduction and bone healing (callus formation/increased mineralization) were observed (Table 3). There were no statistically significant differences in such combined outcomes in relation to the type of radiotherapy (single dose 14/23 vs. fractionated 9/15, *P* = 0.61), type of lesion (pathologic fracture 11/17 vs. impending fracture 12/21, *P* = 0.45) or anatomical location of the lesion (upper extremity 10/14 vs. pelvis 7/12 vs. lower extremity 6/12, *P* = 0.53).

### Survival after treatment

**Figure 5:**
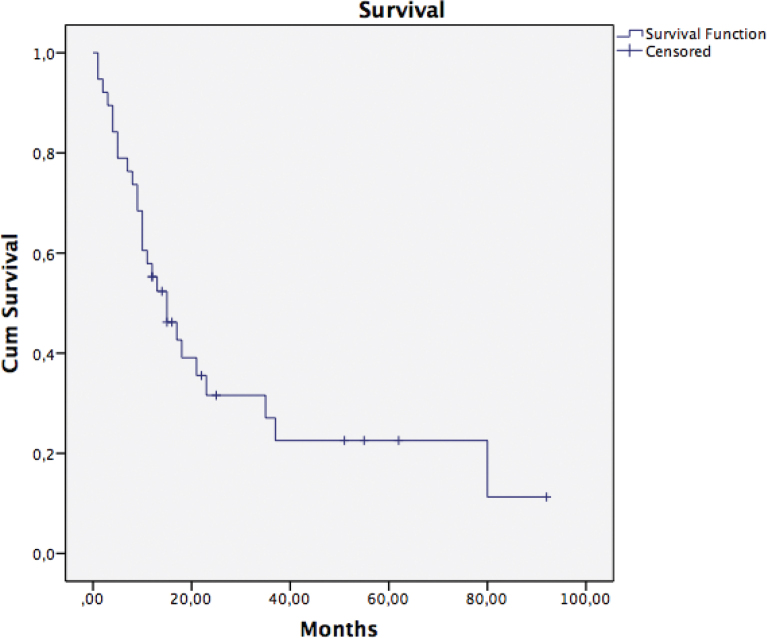
Kaplan-Meier survival curve after the start of treatment with denosumab. The median survival was 15 (IQR: 8–37) months.

The median survival after the start of treatment with denosumab was 15 (IQR: 8–37) months (Figure 5). The median follow-up time for survivors was 22 (IQR: 14–55) months. The 30-day mortality rate was 2/38 and the 1-year survival rate was 21/38.

### Complications and adverse events

Progression from impending fracture to a pathologic fracture occurred in five patients as follows: Three patients (kidney cancer, lung cancer and endometrial cancer) with impending fractures in the proximal femur developed fractures, and all of them underwent surgery in form of internal fixation and osteosynthesis. One patient with bladder cancer had a metastasis in the femoral neck. At the 6-month follow-up after treatment initiation, increased mineralization was observed in the metastatic area, and the patient experienced no pain. Shortly thereafter, a fall trauma resulted in a femoral neck fracture, which was managed surgically by internal fixation. One patient with lung cancer and metastasis located in the sacrum developed a pathologic fracture 6 months after the start of treatment. No additional surgery was performed due to the late palliative state of the patient. No other adverse complications were seen in these five patients.

One patient with prostate cancer developed osteonecrosis of the jaw, which was treated with surgery. None of the patients with upper extremity pathologic or impending fractures needed surgical intervention. No cases of deep vein thrombosis or pulmonary embolism were reported.

## Discussion

We found that combining denosumab with radiotherapy resulted in bone healing and reduced pain in most patients. Our results are in line with the results of the study by Tanaka et al., who reported the efficacy of radiotherapy combined with bone-modifying agents in breast cancer patients with skeletal metastases [13]. They found a significantly greater rate of response to the combination of bone-modifying agents and radiotherapy than to bone-modifying agents alone. However, most patients in their study had received bisphosphonates, and only a few had received denosumab [13]. Like in their study, our patients had osteolytic and mixed osteolytic/osteoblastic bone lesions, but our study included only advanced metastases with impending or pathologic fractures.

Bisphosphonates are osteoclast inhibitors that have been used prophylactically in the early stages of metastatic bone lesions to prevent future skeletal-related events [15]. The binding of bisphosphonates to hydroxyapatite is critical for exerting an anti-osteoclastic effect [16]. Denosumab, on the other hand, works by blocking RANKL [17]. Both osteoblasts and osteoclasts play crucial roles in the bone destruction process in the vicious cycle of bone destruction-tumor growth [18]. We postulate that combined treatment with denosumab and radiotherapy selectively inhibits osteoclastic activity, allowing continued tumor cell-mediated osteoblast activation. This results in remineralization of the tumorous bone, which frequently exceeds the amount of mineralization in surrounding non-tumorous bone. The timing of denosumab administration in relation to radiotherapy may be beneficial in optimizing bone healing. This may be especially important for patients with impending fractures to decrease the risk of pathologic fracture. Higher doses of radiotherapy, such as in some fractionated regimens, may also have an inhibitory effect on osteoblasts and the healing response. However, due to the retrospective nature of our study, we were unable to evaluate these effects.

Patients with bone metastases are fragile and have a high rate of complications and mortality after surgery [19, 20]. Raschka et al. reported a postoperative complication rate of 25%, a mortality rate of 9.3% within the first 30 days and a 1-year survival rate of 52% in a series of 140 patients with skeletal metastases [21]. Wiess et al. reported a 25% postoperative complication rate and a cumulative survival rate of 45% for the first year in a series of 301 patients with skeletal metastases from breast cancer who underwent surgical treatment [22]. In our non-surgical regimen case series, we found a 30-day mortality rate of 5%, a 1-year survival rate of 56%, and a median survival of 15 months. Ehrenstein et al. reported an incidence rate of approximately 6% for osteonecrosis of the jaw in 1,340 patients receiving denosumab with solid metastatic bone lesions over a course of 5 years [23]. We detected osteonecrosis of the jaw in 1/38 patients in this study.

Symptomatic deep vein thromboembolism has been reported in up to 10% of patients with metastatic skeletal lesions undergoing surgical treatment [24]. No patients with symptomatic venous thromboembolism were registered in our series. Previous data have shown that a single dose of 8 Gy is similar to fractionated radiotherapy for relieving pain associated with metastatic skeletal lesions [25]. In our series, we found similar results regarding not only pain relief but also bone healing when combining denosumab with either a single dose of 8 Gy or fractionated radiotherapy. The benefits of single-dose radiotherapy over fractional radiotherapy include, among other benefits, patient convenience and lower costs.

The limitations of this study include its retrospective design, variability in the timing of radiotherapy and denosumab injections and a lack of a control group. In addition, the influence of concurrent antitumoral treatments could not be evaluated. These limitations and especially the small number of patients preclude definitive conclusions.

## Conclusion

Denosumab in combination with radiotherapy appears to be effective in reducing pain and promoting bone healing in patients with pathologic and impending fractures of the pelvis and extremities and may be an alternative to surgery in this group of fragile patients.
